# Expression Profiles of 12 Late Embryogenesis Abundant Protein Genes from *Tamarix hispida* in Response to Abiotic Stress

**DOI:** 10.1155/2014/868391

**Published:** 2014-07-10

**Authors:** Caiqiu Gao, Yali Liu, Chao Wang, Kaimin Zhang, Yucheng Wang

**Affiliations:** ^1^State Key Laboratory of Tree Genetics and Breeding, Northeast Forestry University, 26 Hexing Road, Harbin 150040, China; ^2^Key Laboratory of Forest Genetics & Biotechnology, Nanjing Forestry University, Ministry of Education, Nanjing 210037, China; ^3^Northeast Forestry University Library, 26 Hexing Road, Harbin 150040, China

## Abstract

Twelve embryogenesis abundant protein (*LEA*) genes (named *ThLEA-1 to -12*) were cloned from *Tamarix hispida*. The expression profiles of these genes in response to NaCl, PEG, and abscisic acid (ABA) in roots, stems, and leaves of *T. hispida* were assessed using real-time reverse transcriptase-polymerase chain reaction (RT-PCR). These *ThLEAs* all showed tissue-specific expression patterns in roots, stems, and leaves under normal growth conditions. However, they shared a high similar expression patterns in the roots, stems, and leaves when exposed to NaCl and PEG stress. Furthermore, *ThLEA-1, -2, -3, -4*, and *-11* were induced by NaCl and PEG, but *ThLEA-5, -6, -8, -10*, and *-12* were downregulated by salt and drought stresses. Under ABA treatment, some *ThLEA* genes, such as *ThLEA-1, -2*, and *-3*, were only slightly differentially expressed in roots, stems, and leaves, indicating that they may be involved in the ABA-independent signaling pathway. These findings provide a basis for the elucidation of the function of *LEA* genes in future work.

## 1. Introduction

Adverse environmental conditions, such as salt, drought, and high temperatures, severely limit the growth and geographical distribution of many plants. At the same time, plants have evolved many mechanisms to tolerate these adverse conditions [1–5], and the regulation of gene expression is critical for these processes [6].* Late embryogenesis abundant* (*LEA*) genes play important roles in stress tolerance, especially in drought stress. In wheat, there are several genes which are responsible for drought stress tolerance and produce different types of enzymes and proteins, for instance, late embryogenesis abundant (LEA) protein, responsive to abscisic acid (Rab), rubisco, helicase, proline, glutathione-S-transferase (GST), and carbohydrates during drought stress [7]. The LEA proteins to be firstly identified were from cotton, and they were highly synthesized during the later stages of embryogenesis [8].

To date, hundreds of* LEA*s have been cloned from different plant species [9]. Some* LEA* genes are highly induced by various abiotic or biotic stress treatments including salt [10, 11], drought or osmotic stress [12, 13], heat [14], cold [15, 16], and wounding [17]. In addition, the expression of* LEA* genes are also modulated by abscisic acid (ABA) [10, 18] and ethylene [17]. Transformed plants that overexpress* LEA* genes show improved tolerance to salt stress [19, 20], water deficit or drought conditions [10, 21], and cold [16, 22], but they are hypersensitive to ABA [23].

However, to date the true functions of* LEA*s are not fully understood. Therefore, analyzing the expression patterns of* LEA* genes will be beneficial to understanding their role in stress responses.* Tamarix hispida*, a woody halophyte, is widely distributed in drought-stricken areas and saline or saline-alkali soil in Central Asia and China. This species is highly tolerant to salt, drought, and high temperature, which makes it the desirable tree special to study the salt tolerance mechanism and clone the genes involved in salt tolerance.

In the present study, 12* ThLEA* genes were cloned from* T. hispida*, and the expression patterns of these genes were determined in response to salt (NaCl), drought (PEG), and abscisic acid (ABA) treatments in the roots, stems, and leaves by using real-time reverse transcriptase-polymerase chain reaction (RT-PCR). Our study may provide the fundament data for studying the function of* LEA* genes.

## 2. Materials and Methods

### 2.1. Plant Materials and Treatments

Seedlings of* T. hispida* were grown in pots with a mixture of turf peat and sand (2 : 1 v/v) in a greenhouse at 70–75% relative humidity, light/dark cycle of 14/10 h, and the temperature of 24°C. Two-month-old seedlingswere used for experimental analyses. The seedlings were watered at the roots with one of the following solutions: 0.4 M NaCl, 20% (w/v) PEG_6000_, or 100 *μ*M ABA for 0, 3, 6, 9, 12, and 24 h. In controls, the seedlings were watered with the same volume of fresh water. Following these treatments, the leaves, stems, and roots from at least 20 seedlings were harvested and pooled, frozen immediately in liquid nitrogen, and stored at −80°C for RNA preparation. Three samples were used for real-time RT-PCR biological repeats.

### 2.2. Cloning of the* LEA* Genes from* T. hispida*

In a previous study, 11 cDNA libraries of* T. hispida* treated with NaHCO_3_ were constructed using Solexa technology, which were the 8 libraries, respectively, from roots of* T. hispida* treated with NaHCO_3_ for 0, 12, 24, and 48 h (2 libraries were built from each treatment as biological replication) and 3 libraries from leaves of* T. hispida* treated with NaHCO_3_ for 0, 12, and 24 h. In total 94,359 nonredundant unigenes of >200 nt in length were generated using the SOAP de novo software. Subsequently, these unigenes were searched against the Nr database and the Swiss-Prot database using blastx (http://blast.ncbi.nlm.nih.gov/) with a cut-off *E*-value of 10^−5^ [24, 25]. The unigenes representing the “*LEA*” genes were queried and identified from these libraries according to the functional annotation. Then, primers for the* LEA* genes were designed according to these sequences. RT-PCR and resequencing were used to confirm the* LEA* sequences.

### 2.3. Sequence Alignments and Phylogenetic Analysis

Firstly, the open reading frames (ORFs) of all the* LEA* unigenes (named as* ThLEA-1* to -*12*) were identified using the ORF finder tools from the NCBI (http://www.ncbi.nlm.nih.gov/gorf/gorf.html). Subsequently, the* LEA*s with complete ORFs were subjected to molecular weight (MW) and isoelectronic point (pI) predictions using the Compute pI/Mw tool (http://www.expasy.org/tools/protparam.html). Subcellular location predictions for these genes were undertaken using the Target *P*1.1 Server (http://www.cbs.dtu.dk/services/TargetP/). All the LEA proteins from* T. hispida* and 51 Arabidopsis LEA proteins were subjected to phylogenetic analysis by constructing a phylogenetic tree by the neighbor-joining (NJ) method in ClustalX.

### 2.4. RNA Extraction and Reverse Transcription (RT)

Total RNA was isolated from the roots, stems, and leaves using the CTAB method. Total RNA was digested with DNase I (Promega) to remove any residual DNA. Approximately 1 *μ*g of total RNA was reverse transcribed to cDNA in a 10 *μ*L volume using oligodeoxythymidine and 6-bp random primers following the PrimeScript RT reagent kit (TaKaRa) protocol. The synthesized cDNAs were diluted 10-fold with sterile water and used as templates for real-time RT-PCR.

### 2.5. Quantitative Real-Time RT-PCR

Real-time RT-PCR was carried out in an Opticon machine (Biorad, Hercules, CA) using a real-time PCR MIX Kit (SYBR Green as the fluorescent dye, TOKOBO) and the primers used are shown in Table 1. The* alpha tubulin* (FJ618518),* beta tubulin* (FJ618519), and* actin* (FJ618517) genes were used as internal controls (reference genes) to normalize the total RNA amount present in each reaction. The reaction mixture (20 *μ*L) contained 10 *μ*L of SYBR Green Real-Time PCR Master Mix (Toyobo), 0.5 *μ*M of forward and reverse primer, and 2 *μ*L of cDNA template (equivalent to 20 ng of total RNA). The amplification was performed using the following cycling parameters: 94°C for 30 s, followed by 45 cycles of 94°C for 12 s, 60°C for 30 s, 72°C for 40 s, and then at 81°C for 1 s for plate reading. A melting curve was generated for each sample at the end of each run to assess the purity of the amplified product. Real-time RT-PCR was carried out in triplicate to ensure the reproducibility of the results. The expression levels were calculated from the threshold cycle according to 2^−ΔΔCt^ [26].

## 3. Results

### 3.1. Characterization of* ThLEA*s

In total, 12 unique* ThLEA*s (*ThLEA-1-8*, respectively, with the GenBank numbers KF801660 to KF801667;* ThLEA-9-12*, respectively, with the GenBank numbers KF924555 to KF924558) that are expressed differentially in response to at least one stress were identified from the* T. hispida* cDNA libraries. Among these, eight ThLEA proteins had complete ORFs that encoded deduced polypeptides of 114–584 amino acids with predicted molecular masses of 12.29–65.4 kDa and pIs of 4.75–9.6 (Table 2). Subcellular locations were predicted from protein sequence analysis using the targetP algorithm. The results suggested that ThLEA-3 may be located in the chloroplast, ThLEA-2 and -4 may be secreted proteins, and the locations of the other five* ThLEA* genes with full ORFs may be classified as “other.”

**Figure 1:**
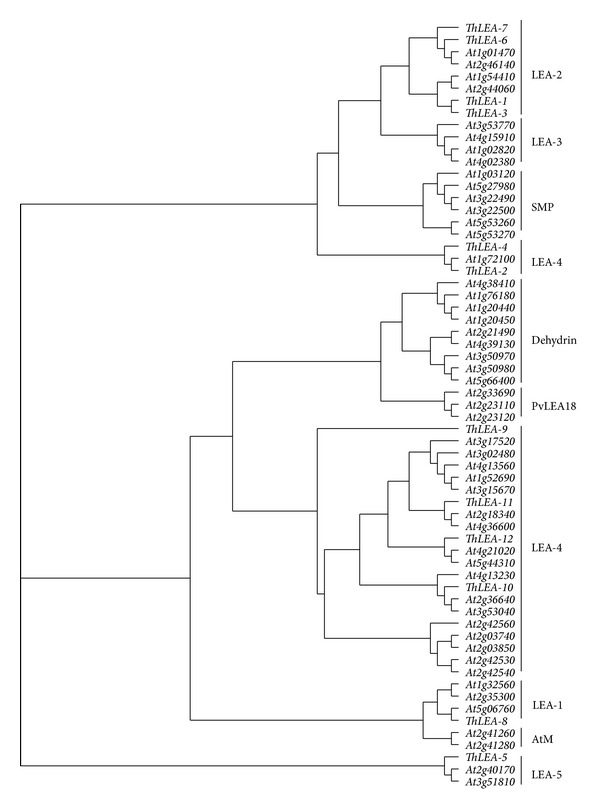
Phylogenetic tree of the 12* ThLEA*s and 51 Arabidopsis* LEA*s based on sequence alignments of the predicted proteins. All the 12 ThLEA proteins from* T. hispida* and 51 Arabidopsis LEA proteins were subjected to phylogenetic analysis by constructing a phylogenetic tree by the neighbor-joining (NJ) method in ClustalX. According to the classification of Hunault and Jaspard [9], the LEA proteins were classified into nine groups.

Hunault and Jaspard [9] identified 51 LEA protein encoding genes in Arabidopsis genome and classified them into nine distinct groups (LEA-1, -2, -3, -4, -5, Atm, SMP, dehydrin, and PvLEA18). According to this classification, the phylogenetic relationships showed that ThLEA-1, -3, -6, and -7 are in the LEA-2 group and ThLEA-2, -4, -9, -10, -11, and -12 are in the LEA-1 group, while ThLEA-8 and -5 are in the Atm and LEA-5 groups, respectively (Figure 1).

### 3.2. Relative Transcript Abundances of* ThLEA*s under Normal Growth Conditions

In order to study the tissue specificity of* ThLEA* gene expression, the relative transcript abundances of the* ThLEA* genes in* T. hispida* roots, stems, leaves, and seeds under normal growth conditions were monitored by real-time RT-PCR. The transcript level of the* actin *gene was assigned as 100, while the transcript levels of* ThLEA* genes were plotted relative to transcript level of* actin* gene (Table 3). The results indicated that* ThLEA-4* is the gene with the highest expression in stems and leaves and in the roots only* ThLEA-10* had greater expression, while the transcript levels of* ThLEA-5 *and* -6* were very low in all four tissues (roots, stems, leaves, and seeds). The expressions of* ThLEA-8* and -*12 *were very high in seeds while those were very low in the other three tissues. In the roots, the transcript level of* ThLEA-6* was lowest and only 0.1 (equivalent to 1/1000 of* actin*). Meanwhile, in stems and leaves, the transcript level of* ThLEA-12* was lowest and less than 1/1000 of* actin*. Moreover, the transcript level of the* ThLEA* genes highly differed among each other and also strongly differed among the four tissues. For instance,* ThLEA-10*, -*11,* and -*12* were expressed mainly in the roots and seeds, and their transcript levels were all exceeded 1220-fold in the seeds.* ThLEA-2* and -*9* were expressed mainly in the stems and seeds, while* ThLEA-1*, -*3,* and -*7* showed relatively high transcript level in the leaves.

### 3.3. The Expression Profiles of* ThLEA*s in Response to NaCl, PEG, and ABA Treatments

#### 3.3.1. NaCl Stress

**Figure 2:**
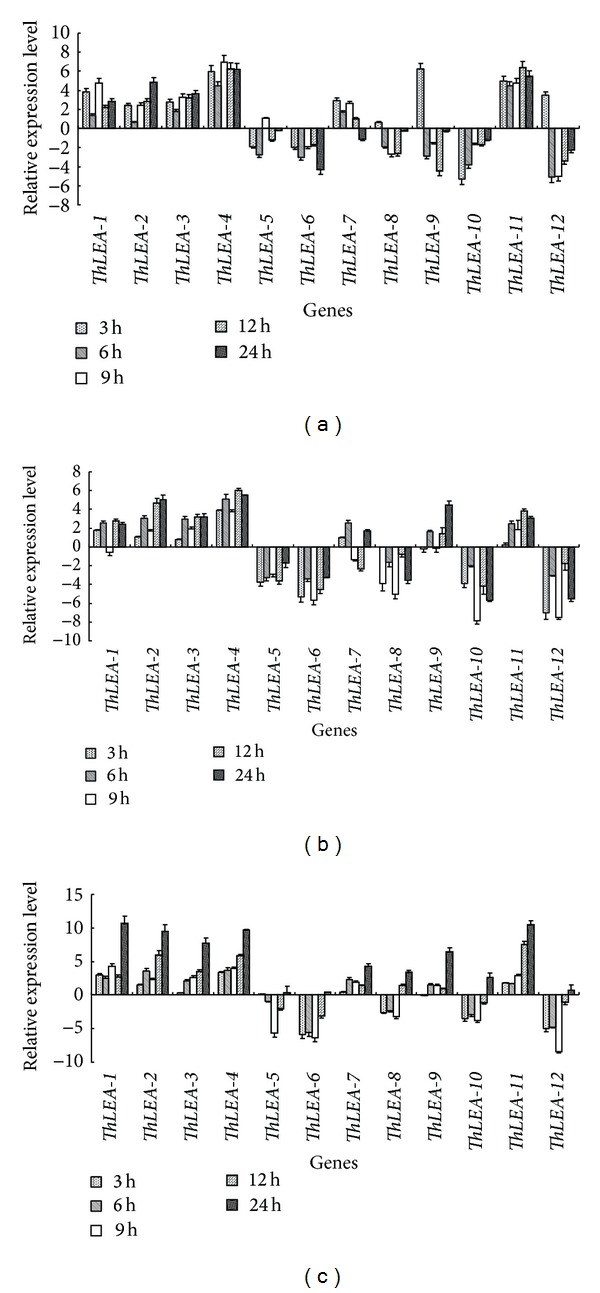
Expression analysis of the 12* ThLEA*s in response to 0.4 M NaCl stress. Relative expression level = transcription level under stress treatment/transcription level under control conditions. All relative expression levels were log_2_ transformed and error bars (SD) were obtained from multiple replicates of the real-time RT-PCR. (a), (b), (c): expression of* ThLEA*s in roots, stems, and leaves, respectively.

The transcript patterns of all of the* ThLEA* genes were quite similar among the roots, stems, and leaves under NaCl stress. Furthermore,* ThLEA*-*2*, -*3*, -*4,* and -*11* were induced (>2-fold) during NaCl stress period.* ThLEA-7* was also upregulated during most treatment period, and six* ThLEA* genes reached their peak expression level in the leaves at 24 h of stress. The most highly induced gene was* ThLEA-1*, which was induced 1596-fold. However,* ThLEA-5, -6*,* -8*,* -10,* or -*12* were highly downregulated at almost each stress time point. Except for* ThLEA-5*, the other four downregulated genes reached lowest transcript levels at 9 h in stems and leaves. Interestingly, distinct from these genes, the transcript pattern of* ThLEA-9* was different among the three tissues. In the roots, it was induced at 3 h but downregulated at the other time points, while in stems and leaves it was mainly upregulated. Similar with the other upregulated* ThLEA*s,* ThLEA-9* reached greatest expression at 24 h in the leaves (Figure 2).

#### 3.3.2. PEG Stress

**Figure 3:**
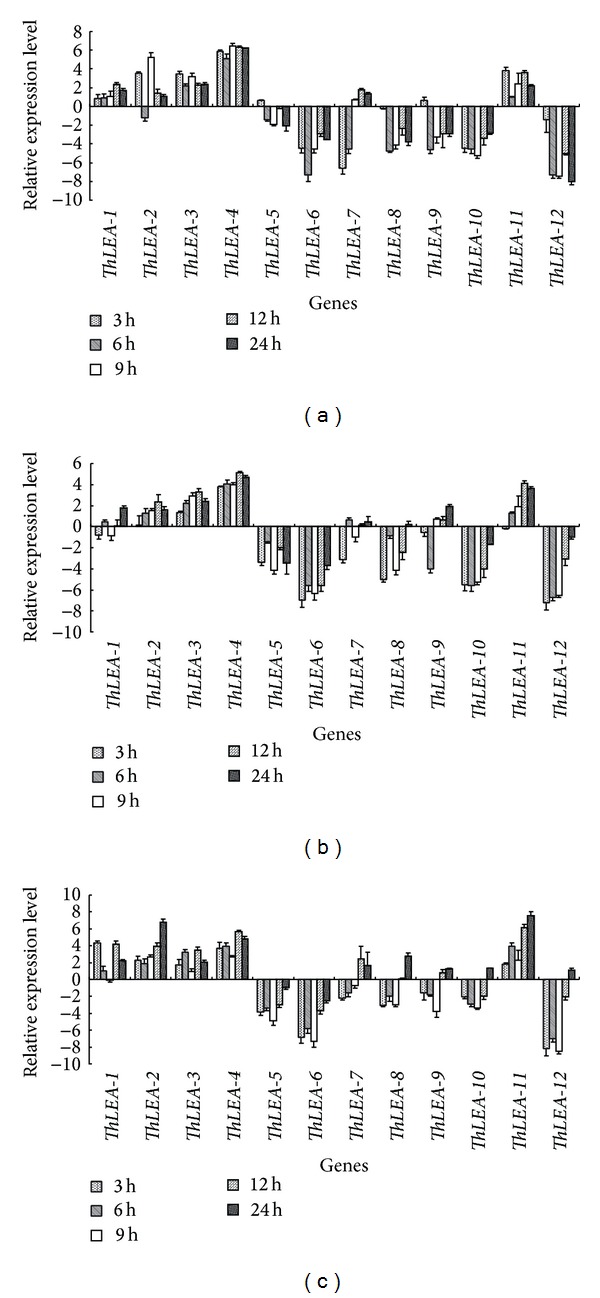
Expression analysis of the 12* ThLEA*s in response to 20% PEG6000 stress. Relative expression level = transcript level under stress treatment/transcript level under control conditions. All relative expression levels were log_2_ transformed and error bars (SD) were obtained from multiple replicates of the real-time RT-PCR. (a), (b), (c): expression of* ThLEA*s in roots, stems, and leaves, respectively.

Consistent with NaCl stress, the transcript patterns of these* ThLEA *genes under PEG stress were divided into two distinct groups (Figure 3). One group was composed of* ThLEA-1*,* -2*,* -3*,* -4, *and -*11*, and these genes were mainly upregulated. The second group included* ThLEA-5*, -*6*, -*8*, -*9*, -*10*, and -*12* that were largely downregulated during the PEG treatment period.

#### 3.3.3. ABA Treatment

**Figure 4:**
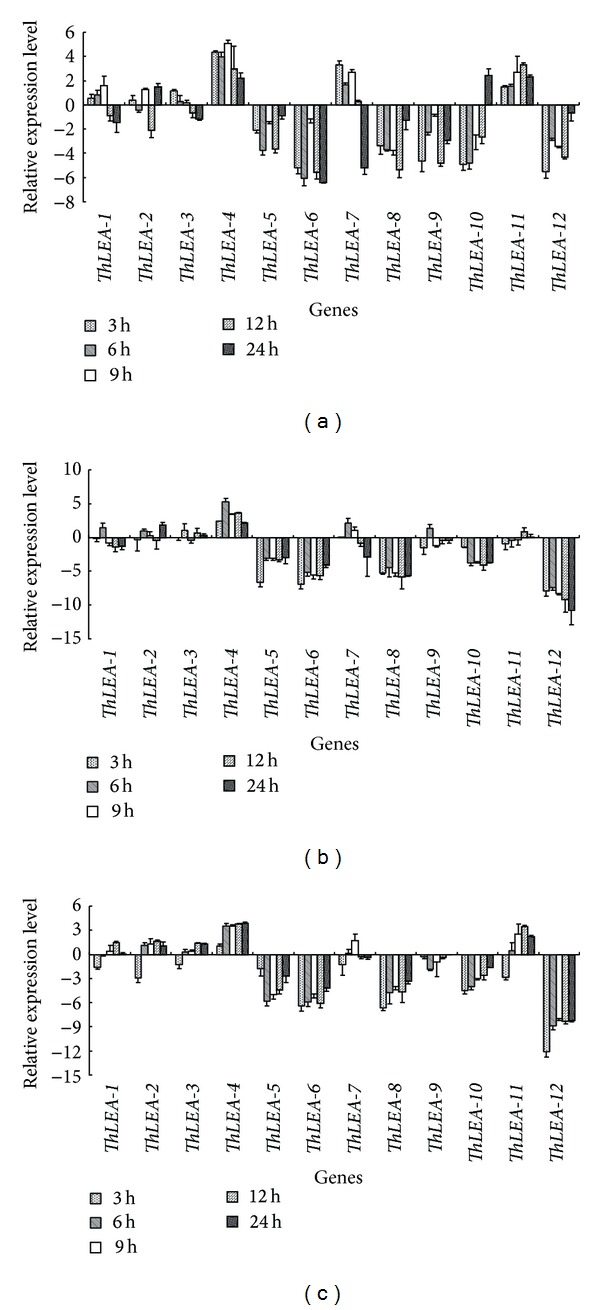
Expression analysis of the 12* ThLEA*s in response to 100 *μ*M ABA treatment. Relative expression level = transcript level under stress treatment/transcript level under control conditions. All relative expression levels were log_2_ transformed and error bars (SD) were obtained from multiple replicates of the real-time RT-PCR. (a), (b), (c): expression of* ThLEA*s in roots, stems, and leaves, respectively.

After application of exogenous ABA,* ThLEA-1*, -*2,* and -*3* were only slightly differentially regulated in the roots, stems, and leaves, indicating that they may be regulated via the ABA-independent signaling pathway. However, the genes that were downregulated by PEG and/or NaCl stress were also highly downregulated by ABA treatment. In contrast,* ThLEA-4*,* -7*, and* -11* were mainly upregulated, especially* ThLEA-4*, whose transcript levels in the stem at 6 h were increased by 38-fold compared with the control (Figure 4).

## 4. Discussion

In the present study, 12* ThLEA* genes were identified from* Tamarix* RNA-seq data base, and the transcript abundance of each gene was assessed in normal growth conditions by real-time RT-PCR. Furthermore, NaCl and PEG were used to simulate salinity and drought conditions, respectively, and the expression levels of the* ThLEA* genes were investigated in roots, stems, and leaves in response to these stress environments.

Many studies have shown that members of the plant* LEA* family displayed tissue-specific expression and are involved in various processes in plant growth and development. For example, in Arabidopsis, 22 of the 51* LEA* genes (43%) showed high expression levels (relative expression >10) in the nonseed organs in the absence of a stress or hormone treatment [27]. The* OsLEA3-2* gene is not expressed in vegetative tissues under normal conditions and it is thought to play an important role in the maturation of the embryo [10]. Moreover, the transcripts of* MsLEA3-1* were strongly enriched in leaves compared with roots and stems of mature alfalfa plants [20]. Expression profiles of the 12* ThLEA* genes in different tissues of* T. hispida* were determined under normal growth conditions and this showed that most of the* ThLEA*s were expressed in various organs and tissues. However, they displayed different expression levels between the different tissue types and this may reflect the complexity of functions performed by this gene family [28]. Among the 12* ThLEA*s,* ThLEA-10*, -*11*, and -*12* were mainly expressed in the roots, suggesting that these genes may play roles in root stress response.* ThLEA-2* and -*9* were highly expressed in the stem, while* ThLEA-1*, -*3*, and -*7* may play important roles in the leaves as these genes are highly and specifically expressed in leaves. Moreover,* ThLEA-4* has very high expression level in the roots, stems, and leaves, suggesting that it might play important roles in all of these tissues. The expression levels of* ThLEA-5*, -*6*, -*8*, and -*12* were very low in each of the tissues examined, indicating that these genes may play a relatively unimportant role in these tissues under normal growth conditions.

There is increasing evidence suggesting that* LEA* genes are associated with abiotic stress tolerance, particularly in plant responses to dehydration, salt, and cold stresses [18, 29]. In Arabidopsis, of the 22 genes highly expressed in nonseed tissues, 12 were induced by more than 3-fold in response to cold, drought, and salt stresses [27]. In sweet potato plants,* IbLEA14* expression was strongly induced by dehydration and NaCl [30]. In the present study, half of the 12* ThLEA* genes (*ThLEA-1*, -*2*, -*3*, -*4*, -*7*, and -*11*) were induced under salt and drought stress, indicating that these genes might play important roles in response to salt and drought stresses in* T. hispida*. Bies-Ethève et al. [31] reported that* LEA* genes from the same group do not show identical expression profiles and regulation of* LEA* genes that show apparently similar expression patterns does not systematically involve the same regulatory pathway. Consistent with this, the six upregulated* ThLEA*s in* T. hispida* belong to two* LEA*s groups (*LEA-2* and* LEA-4*), while* LEA*s in the same group (such as* LEA-4*) showed completely different expression patterns.

There are ABA-dependent and ABA-independent regulatory networks controlling plant responses to abiotic stresses.* LEA* genes are often considered as being ABA-regulated and previous studies have shown that some* ThLEA* genes can be induced by exogenous ABA [10, 30]. However, our study shows that some* ThLEA* genes, including* ThLEA-1*, -*2*, and -*3*, were highly induced by abiotic stresses but were not regulated by ABA. Consistent with this, Bies-Ethève et al. [31] reported that most of the* LEA* genes in Arabidopsis seedlings show no response at all following exogenous ABA treatment. Hundertmark and Hincha [27] deduced the presence of different signal transduction pathways in different Arabidopsis tissues (vegetative plants and seeds) by comparing the expression of* LEA *genes after ABA treatment. In contrast, we found that expression patterns were highly similar in the three tissues examined after ABA treatment. Expression analyses showed that nine* ThLEA* genes in three tissues of* T. hispida* were upregulated or downregulated by ABA, which suggests that these* ThLEA* genes are regulated by ABA-dependent stress resistance pathways. These findings demonstrate that the* ThLEA* genes involved in salt and drought stress resistance in* T. hispida* are probably regulated by two different pathways (ABA-dependent and ABA-independent). Further studies are required to elucidate the functions of the* ThLEA*s in response to the abiotic stress and to fully characterize the signaling pathways that regulate their expression.

## 5. Conclusion

In summary, we identified 12* ThLEA* genes from* T. hispida*, which belong to three groups. The LEA-1 group includes ThLEA-2, -4, -9, -10, -11, and -12 proteins, the LEA-2 group contains ThLEA-1, ThLEA-3, ThLEA-6, and ThLEA-7, and ThLEA-8 and -5 are in the Atm and LEA-5 groups. The* ThLEA* genes displayed tissue-specific expression patterns under normal growth conditions.* ThLEA-10*, -*11*, and -*12* were expressed mainly in the roots and seeds.* ThLEA-2* and -*9* were preferentially expressed in the stems and seeds, while* ThLEA-1*, -*3*, and -*7* showed high transcript level in the leaves. Furthermore, real-time RT-PCR analysis showed that the* ThLEA* genes were regulated by salt and drought stresses.* ThLEA-1*,* -2*,* -3*,* -4*,* -7*, and* -11* were mainly induced by salt and drought stress. But* ThLEA-5*, -*6*, -*8*, -*9*, -*10*, and -*12* were largely downregulated during the NaCl and PEG treatment. After the application of exogenous ABA, the expression levels of all* ThLEA* genes (except for the* ThLEA-1*,* -2*, and* -3*) were obviously different under the ABA treatment. Our studies will contribute to a better understanding of the functions of* ThLEA* genes involved in stress response in* T. hispida*.

## Figures and Tables

**Table 1:** Primers used for quantitative real-time RT-PCR analysis.

Gene	GenBank accession number	Forward primer (5′-3′)	Reverse primer (5′-3′)
*ThLEA-1 *	KF801660	CAGCGAAGTTTGGATGGAATG	ACCTGTCGCCAATCAGAAGAT
*ThLEA-2 *	KF801661	CACACAATAGAAACCGTAGAG	CCTCCATGGTCTCCTTAACT
*ThLEA-3 *	KF801662	CGAGATCCTTGGTGGAGCTCG	TCACGTATGTGTCATGACTAC
*ThLEA-4 *	KF801663	GTGGCTATCTCGATTCGTAGT	GCAACAGACACCAACCAAGAT
*ThLEA-5 *	KF801664	GTTCGCAGCAGGAAAGAGCAG	ACTCCGTCAACACGGTACTACT
*ThLEA-6 *	KF801665	GTTGAGGATGTGGATATCAAG	CGAGTTCATAATCGATATCC
*ThLEA-7 *	KF801666	TCATGATATTGGTGAGAAG	AGTGGTATCTTAACAGTCTCT
*ThLEA-8 *	KF801667	GATGACGACACATGATGAAGC	AGCCTCCACAGTCCCTTCCGT
*ThLEA-9 *	KF924555	GTGGTTGTGGAGATGAACGGAG	GGTAGCAGCATCATCAGCAATG
*ThLEA-10 *	KF924556	AGAGTCAGCAACCGATACAGC	TCTCTGCCTTGCTGATTCCTC
*ThLEA-11 *	KF924557	ACGGAGGAAGCTAGGCACGAAG	CTGCTGCGTCCTTGTATTCC
*ThLEA-12 *	KF924558	CAAGATGCAGGAGGTGGTGAT	ATCTGCTATTCTTCTGCCTGT
*Actin *	FJ618517	AAACAATGGCTGATGCTG	ACAATACCGTGCTCAATAGG
*α-Tubulin *	FJ618518	CACCCACCGTTGTTCCAG	ACCGTCGTCATCTTCACC
*β-Tubulin *	FJ618519	GGAAGCCATAGAAAGACC	CAACAAATGTGGGATGCT

**Table 2:** Characteristics of the 8 *ThLEAs* from *T. hispida* that had full ORFs.

Gene name	Deduced number of amino acids	MW (kDa)	pI	Predicted subcellular localization∗
*ThLEA-1 *	584	65.40	8.18	Other
*ThLEA-2 *	435	46.70	8.67	Secreted
*ThLEA-3 *	462	51.31	8.02	Chloroplast
*ThLEA-4 *	212	23.55	9.60	Secreted
*ThLEA-5 *	114	12.29	5.54	Other
*ThLEA-6 *	151	16.31	4.97	Other
*ThLEA-7 *	318	35.31	4.75	Other
*ThLEA-8 *	171	17.49	8.02	Other

*Subcellular localization was predicted from protein sequence analysis using the targetP algorithm.

**Table 3:** Relative transcript abundance of the 12 *ThLEA*s in different tissues of *T. hispida.* The transcript levels of the 12 *ThLEA* genes were plotted relative to expression of the *actin* gene. Transcript levels of the *actin* gene in roots, stems, leaves, and seeds were all assigned as 100.

Gene	Relative abundance			
	Roots	Stems	Leaves	Seeds
*ThLEA-1 *	6.8	11.6	36.2	10
*ThLEA-2 *	5.3	26.4	12.1	230
*ThLEA-3 *	8.2	23.2	64.8	4
*ThLEA-4 *	188.1	365.6	355.5	161
*ThLEA-5 *	1.5	0.2	0.3	20
*ThLEA-6 *	0.1	1.4	0.7	3
*ThLEA-7 *	0.8	19.5	27.9	10
*ThLEA-8 *	8.5	0.8	1.5	770
*ThLEA-9 *	17.7	61.7	26.2	180
*ThLEA-10 *	198.3	2.0	5.1	1240
*ThLEA-11 *	161.8	4.1	23.7	6050
*ThLEA-12 *	9.9	0.1	0.1	1220
